# Atrophic Cirrhosis of the Liver*Delivered in the Clinical Amphitheatre of the Medico-Chirurgical Hospital.

**Published:** 1907-12

**Authors:** John V. Shoemaker

**Affiliations:** Professor of Materia Medica, Therapeutics, Clinical Medicine, and Diseases of the Skin in the Medico-Chirurgical College and Hospital of Philadelphia


### TEXAS MEDICAL JOURNAL.

Established July, 1885.

F. E. DANIEL, M. D., - - - - - Editor, Publisher and Proprietor

PUBLISHED MONTHLY.—SUBSCRIPTION $1.00 A YEAR.

VOL. XXIII. AUSTIN, DECEMBER, 1907. No. 6.

The publisher is not responsible for the views of contributors.

For the Texas Medical Journal.

### Atrophic Cirrhosis of the Liver.*

BY JOHN V. SHOEMAKER, M. D., LL. D.,
Professor of Materia Medica, Therapeutics, Clinical Medicine, and Diseases of the Skin in the Medico-Chirurgical College and Hospital of Philadelphia.

The next patient—47 years of age—presents typical clinical symptoms, and to assist you in arriving at an absolutely correct diagnosis you must consider his history carefully as regards the etiology and diagnosis of his trouble. The history reads as follows:

*Family History.*—The father died at the age of 47 years from asthma, and the mother at the age of 65 years from "heart failure." He has five brothers and one sister living and well. The history is entirely negative as regards cancer and tuberculosis.

*Previous Personal History.*—As a child he had measles, whooping cough and mumps. At the age of 12 years he had gastric catarrh, but claims that he was then entirely cured. Since then he has always enjoyed good health until his present trouble.

*Social History.*—He is married and is the father of three children. One child died from spinal meningitis following an injury. The two children living and the wife are in good health.

By occupation he is a printer.

*Habits.*—His custom is to drink one or two cups of coffee or tea with each meal. He chewed tobacco excessively until two years ago. His usual custom was to drink from four to five glasses of whisky daily until last February, one month ago, when he discontinued drinking it.

*Present Illness.*—Two weeks ago he developed an attack of influenza with sore throat. He remained in bed, but suffered only

*Delivered in the Clinical Amphitheatre of the Medico-Chirurgical Hospital.

a slight pain in the abdomen. He noticed that his urine was decreasing in quantity and was highly colored. His stools were much lighter in color and the bowels were constipated. This condition was followed by a gradual increase in size of his abdomen until now he measures 39 inches around the waist. He also complains of dyspnea and cough, especially so on exertion.

*Physical Signs.*—The patient's skin is slightly icteroid in color; so are the conjunctivæ of both eyes. The eyes react normally to both light and distance.

The abnormal signs elicited over the chest is a soft blowing murmur, diastolic in time, over the aortic area. Percussion reveals a slight increase of the precordial area, the apex beat is felt but not visible a half inch to the left of the nipple line.

Owing to the marked distension of the abdomen the liver can not be palpated, but percussion produces a dull note in the mid-axillary line in the seventh interspace, showing that the area of dullness over the liver is diminished. In the flanks on percussion a dull or rather flat note is elicited in the recumbent position and a rather tympanitic note over the anterior portion of the abdomen. In the sitting posture a dull note on percussion is elicited below the umbilicus. The blue lines over the abdomen are due to the dilatation and engorgement of the veins, the cause of which is due to the establishment of compensatory circulation of the venous system by means of the superficial epigastric and internal mammary veins, forming around the umbilicus the characteristic "caput medusa," as you may notice is present in this patient.

The feet around the ankles are edematous and pit on pressure.

*Chief Complaints.*—Dyspnea on exertion, anorexia, itching of the skin, malaise and chronic constipation.

A urinalysis has been made on three different occasions, and the findings have always been as follows:

Albumin—Negative.
Glucose—Negative.
Indican—Reaction slight.
Specific Gravity—1020.
Reaction—Alkaline.
Appearance—Clear.

*Microscopic Examination of the Sediment.*

Casts—Negative.
Cylindroids—A few.
Phosphate Crystals—A few.

*Diagnosis.*—The diagnosis is usually based upon the history as regards the probable cause, such as alcoholism, gout, diabetes, rheumatism, and probably syphilis; the onset, the physical signs, and by elimination of other internal organs being involved, such as the heart, lung, spleen and kidneys.

This disease may be mistaken for chronic peritonitis with effusion, but the tabulated outline on the blackboard will help you in differentiating the two diseases.

*Atrophic Cirrhosis.*

1. Cause due to alcoholism.
2. No history of acute attacks.
3. No pain on pressure.
4. No elevation of temperature.
5. Lungs not affected.
6. Hepatic facies present.
7. "Caput medusa" present.
8. Abdominal veins enlarged.

*Peritonitis With Effusion.*

1. Caused by tubercular infection.
2. History of acute attacks.
3. Pain on pressure.
4. Elevation of temperature.
5. Usually tubercular infection of the lungs.
6. Hepatic facies absent.
7. "Caput medusa" present.
8. Abdominal veins not enlarged.

*Pathology.*—This form of cirrhosis of the liver is commonly known as "hob-nailed liver"; so called because on the surface of the liver are seen grayish-white bands of connective tissue surrounding yellowish areas which project above the surface by the compression due to the formation of the connective tissue. On section these areas resist the cutting knife. The capsule of the liver is much thickened and very fibrous, causing a reduction in the size of the liver, which becomes hard and granular. Sections of the liver under the microscope show a proliferation of connective tissue cells around the terminal branches of the portal veins compressing them together with the hepatic cells and thus obstructing the portal circulation through the liver.

The hepatic cells are seldom changed but the biliary canaliculi are, as a rule, increased in number.

*Etiology.*—The cause of the cirrhosis is undoubtedly due to alcoholism, which is really the most frequent cause in all forms of cirrhosis of the liver, and especially in the atrophic form. The more alcohol contained in the beverage used the more apt and the sooner is the disease developed. Alcoholic drinks taken before meals on an empty stomach has its influence in producing the disease more rapidly, the alcohol being absorbed more rapidly in an empty stomach than when the stomach is partly filled with food. Among the other predisposing causes are the chronic diseases which favor connective tissue formation, such as syphilis, gout, tuberculosis and carcinoma. Age and the male sex have their influence. The disease seldom occurs before middle life.

*Treatment.*—This patient, owing to the large amount of fluid in his abdominal cavity, is obliged to remain in bed. His diet consists of milk and broths; no solid food or anything that tends to irritate the gastro-intestinal canal should be given this patient. To keep his skin active, he receives frequent warm baths, followed by an alcohol rub. He is inclined to be constipated, hence we first gave him a grain and a half of calomel in broken doses, followed by a saline purge. Carthársis, diuresis and diaphoresis are all indicated, hence we have prescribed the pill containing:

℞ Pulveris scillæ
Massæhydrargyri
Pulveris digitalis aa, gr. i
Misce fiat pilula No. 1.
Sig.: One such pill every four hours.

This combination is known as Niemyer's pill, and is exceedingly valuable in these cases. It has decreased the ascitic condition somewhat, but should medicinal agent fail to reduce the ascites materially we will resort to *paracentesis abdominis* to draw off the fluid. After all necessary aseptic precautions are taken and the bladder is emptied, the patient is placed in the sitting posture and the trocar is quickly thrust through the already anesthetized area over the linea alba about three inches above the pubis. The trocar should not be inserted more than one inch. After the proper amount of fluid is drawn off, gradually the wound is covered over with a small thin pledget of cotton saturated with collodion. Pressure may be made over the abdomen by a proper binder, but it has little or no value.

*Prognosis.*—All of these patients eventually die from this disease. In a few cases life may be prolonged, and they may die of some intercurrent disease before the cirrhotic condition has developed sufficiently to cause death.

